# An Improved Adaptive Compensation H∞ Filtering Method for the SINS’ Transfer Alignment Under a Complex Dynamic Environment

**DOI:** 10.3390/s19020401

**Published:** 2019-01-19

**Authors:** Weiwei Lyu, Xianghong Cheng, Jinling Wang

**Affiliations:** 1 School of Instrument Science & Engineering, Southeast University, Nanjing 210096, China; lvww0220@seu.edu.cn; 2 Key Laboratory of Micro-Inertial Instrument and Advanced Navigation Technology, Ministry of Education, Southeast University, Nanjing 210096, China; 3 School of Civil and Environmental Engineering, University of New South Wales, Sydney, NSW 2052, Australia; jinling.wang@unsw.edu.au

**Keywords:** strapdown inertial navigation system (SINS), transfer alignment, filtering divergence, adaptive compensation, robustness factor, complex dynamic environment

## Abstract

Transfer alignment on a moving base under a complex dynamic environment is one of the toughest challenges in a strapdown inertial navigation system (SINS). With the aim of improving rapidity and accuracy, velocity plus attitude matching is applied in the transfer alignment model. Meanwhile, the error compensation model is established to calibrate and compensate the errors of inertial sensors online. To suppress the filtering divergence during the process of transfer alignment, this paper proposes an improved adaptive compensation H∞ filtering method. The cause of filtering divergence has been analyzed carefully and the corresponding adjustment and optimization have been made in the proposed adaptive compensation H∞ filter. In order to balance accuracy and robustness of the transfer alignment system, the robustness factor of the adaptive compensation H∞ filter can be dynamically adjusted according to the complex external environment. The aerial transfer alignment experiments illustrate that the adaptive compensation H∞ filter can effectively improve the transfer alignment accuracy and the pure inertial navigation accuracy under a complex dynamic environment, which verifies the advantage of the proposed method.

## 1. Introduction

Initial alignment is vital to a strapdown inertial navigation system (SINS) as its performance is largely decided by the accuracy and rapidity of the alignment process [1,2,3]. Since the initial velocity and position can be easily obtained by a reference navigation system, such as a global navigation satellite system (GNSS), initial alignment mainly refers to determining the SINS’ initial attitude information [4,5]. According to different alignment modes, the initial alignment methods can be categorized into self-alignment, combination alignment, and transfer alignment. Compared with self-alignment and combination alignment, transfer alignment is more rapid, and alignment time can be greatly shortened [2,6,7]. Moreover, transfer alignment can achieve high alignment accuracy of slave SINS (S-SINS) when the device accuracy of master SINS (M-SINS) is high [6,8]. 

Based on the above advantages, transfer alignment has been widely used in various applications, such as aircraft and ships [1,2,3,4,5,6,7,8,9,10,11]. In the transfer alignment of airborne distributed position and orientation systems (DPOS), Gong et al. [9] utilized an unscented Kalman filter (UKF) to estimate the flexural angles and flexural lever arm variations. Moreover, to deal with structural flexibilities between M-SINS and S-SINS when conducting transfer alignment, an extended Kalman filter (EKF) is often used in large vehicles [12] and flexible aircrafts [13] to estimate the motion states. With the purpose of realizing rapid and accurate transfer alignment for an airborne weapon’s SINS, a mixed H2/H∞ filtering algorithm was designed to deal with the observation noise, which contains both Gaussian white noise and energy limited noise [3]. In [1], the index of alignment degree was put forward to attempt an engineering application of transfer alignment accuracy evaluation and identification. To overcome the lack of real-time information on dynamic flexure characteristics in shipboard transfer alignment, a parameter estimation algorithm was proposed to utilize the angular increment difference measured by the M-SINS and the S-SINS [8]. Cheng et al. [10] put forward a new algorithm of “velocity plus attitude” transfer alignment based on a modified adaptive filter to solve the problem that the performance of shipborne SINS transfer alignment is affected by the system dynamic model and noise statistical properties. Overall, with regards to different application objects and application environments of transfer alignment, a lot of work has been done. However, when the environment is complex and dynamic, realizing rapid and accurate transfer alignment is still a very challenging problem.

When the mathematical model or the priori information on the external noise is not exactly known or is unavailable in the transfer alignment system, the Kalman filtering scheme is no longer applicable [14,15,16]. In contrast, the H∞ filter does not make assumptions about the statistical characteristics of the input signal, and it has good robustness against the model uncertainties, such as measurement noise, external disturbance, and structural uncertainty [17,18,19,20]. The main purpose of the H∞ filter is to design an estimator to minimize the H∞ norm of the error system in order to ensure that the L2-induced gain from the disturbance input to the filter error output is less than a prescribed level [14,21,22,23]. Hence, the H∞ filter has more advantages than the Kalman filter under a complex dynamic environment, and it is appropriate in many practical applications [14,20,24,25,26,27]. In [28], a fuzzy adaptive nonlinear H∞ filter was proposed for the unmanned aerial vehicle (UAV) localization problem. In [15], an exponentially stable robust filter was designed to restrain the impact from uncertain disturbances to estimation errors in the micro-electro-mechanical system-based INS/global positioning system (GPS) integrated navigation system. In order to make full use of the delayed information, a robust weighted H∞ filter was defined for networked systems with intermittent measurements under the discrete-time framework [29]. To improve the accuracy and robustness of the state estimation when the noise statistics are not known a priori, Jia et al. [30] combined the H∞ filter with a sparse-grid quadrature (SGQ) filter. In [20], an adaptive H∞ filtering method, which can estimate and compensate time delay online and adaptively adjust the robust factor according to the unknown external environment, was designed. However, there are few studies on suppressing the divergence of the H∞ filter. If there is abnormal interference that does not meet the convergence condition of the H∞ filter in practical systems, the filtering accuracy will inevitably decrease [25]. Therefore, the robust filtering methods, which can effectively suppress filtering divergence in practical systems, are worth further study.

In this paper, the transfer alignment problem on a moving base under a complex dynamic environment is studied. The SINS error dynamics model, velocity plus attitude matching measurement model, and the sensor error compensation model are established for the transfer alignment. To improve the rapidity and accuracy of transfer alignment under a complex dynamic environment, an adaptive compensation H∞ filtering method is designed. The proposed filtering method can effectively suppress the filtering divergence in transfer alignment, and the robustness factor of the filter can be dynamically adjusted with the change of the external environment. The aerial transfer alignment experiments verified the effectiveness of the proposed improved method, and the alignment accuracy is significantly improved.

The rest of this paper is organized as follows: In Section 2, the transfer alignment model—which includes SINS error dynamics model, velocity plus attitude matching measurement model, and the sensor error compensation model—is established. The H∞ filtering method is presented in Section 3. In Section 4, an adaptive compensation H∞ filtering method for SINS’ transfer alignment is proposed and analyzed in detail. In Section 5, seven groups of aerial transfer alignment experiments are conducted to verify the validity of the proposed method. Finally, the conclusions are drawn in Section 6.

## 2. Transfer Alignment Model

### 2.1. SINS Error Dynamics Model

In this paper, “east–north–up (ENU)” is selected as the navigation frame, and “right–forward–up” is selected as the body frame. The SINS processes the output of inertial components based on mathematical equations to obtain the navigation data. The velocity error equation describes the analytical relationship between the accelerometer output and the carrier’s velocity [5,6]. It can be expressed in the navigation frame *n* as follows:

(1) $$\delta {\dot{V}}^{n}=f^{n}\times \phi^{n}-(2\omega_{ie}^{n}+\omega_{en}^{n})\times \delta V^{n}-(2\delta \omega_{ie}^{n}+\delta \omega_{en}^{n})\times V^{n}+\nabla^{n}$$

where $\delta V^{n}={\left[\begin{array}{ccc} \delta V_{E}^{n} & \delta V_{N}^{n} & \delta V_{U}^{n} \end{array}\right]}^{T}$ represents the linear velocity errors; $\delta {\dot{V}}^{n}$ is the derivative of $\delta V^{n}$; $f^{n}={\left[\begin{array}{ccc} f_{E}^{n} & f_{N}^{n} & f_{U}^{n} \end{array}\right]}^{T}$ represents the specific force vector; $\nabla^{n}={\left[\begin{array}{ccc} \nabla_{E}^{n} & \nabla_{N}^{n} & \nabla_{U}^{n} \end{array}\right]}^{T}$ is the three-axis accelerometer bias in the navigation frame; $\omega_{ie}^{n}$ is the Earth’s rotation rate; $\delta \omega_{ie}^{n}$ is the derivative of $\omega_{ie}^{n}$; $\omega_{en}^{n}$ is the angular rate of the earth frame relative to the navigation frame; and $\delta \omega_{en}^{n}$ is the derivative of $\omega_{en}^{n}$. $\omega_{ie}^{n}$ and $\omega_{en}^{n}$ can be expressed by Equations (2) and (3), respectively:

(2) $$\omega_{ie}^{n}={\left[\begin{array}{ccc} 0 & \omega_{ie}\cos L & \omega_{ie}\sin L \end{array}\right]}^{T}$$

(3) $$\omega_{en}^{n}={\left[\begin{array}{ccc} -\frac{V_{N}}{R_{M}} & \frac{V_{E}}{R_{N}} & \frac{V_{E}\tan L}{R_{N}} \end{array}\right]}^{T}$$

where *R_M_* is the radius of curvature in meridian, *R_N_* is the radius of curvature in prime vertical, and *L* is the latitude.

The position error equations of SINS can be expressed as follows [9]:

(4) $$\delta \dot{L}=\frac{\delta V_{N}^{n}}{R_{M}}$$

(5) $$\delta \dot{\lambda }=\frac{\delta V_{E}^{n}}{R_{N}}\sec L+\delta L\frac{V_{E}^{n}}{R_{N}}\tan L\sec L$$

where *δL* and *δλ* are the latitude error and longitude error.

The attitude angle error of SINS is influenced by instruction angular velocity and gyro bias. In addition, gyro bias causes the growth of attitude angle error in the opposite direction. The attitude error equation in the navigation frame *n* can be expressed as follows [9,10]:

(6) $${\dot{\phi }}^{n}=\phi^{n}\times \omega_{in}^{n}+\delta \omega_{in}^{n}-\varepsilon^{n}$$

where $\phi^{n}={\left[\begin{array}{ccc} \phi_{E}^{n} & \phi_{N}^{n} & \phi_{U}^{n} \end{array}\right]}^{T}$ is the attitude angle error of the calculated platform represented in the local ENU navigation frame; $\varepsilon^{n}={\left[\begin{array}{ccc} \varepsilon_{E}^{n} & \varepsilon_{N}^{n} & \varepsilon_{U}^{n} \end{array}\right]}^{T}$ is the three-axis gyro drift in the navigation frame; $\omega_{in}^{n}$ is the angular rate of the inertial frame relative to the navigation frame; and $\delta \omega_{in}^{n}$ is the derivative of $\omega_{in}^{n}$. Here, $\omega_{in}^{n}$ can be obtained using the following equation:

(7) $$\omega_{in}^{n}=\omega_{ie}^{n}+\omega_{en}^{n}$$

In system equations, the three-axis accelerometer bias and the three-axis gyro drift are assumed not to change over time [4,10]. Thus,

(8) $${\dot{\nabla }}^{b}=0$$

(9) $${\dot{\varepsilon }}^{b}=0$$

The model used in Equations (8) and (9) is a model of a random constant that corresponds to a model of an exponentially correlated process when time is endless. In the model of transfer alignment, twelve dimensions’ states are selected to establish the equation of states. The system’s states can be expressed as follows:

(10) $$X(t)={\left[\delta L,\delta \lambda ,\delta V_{E},\delta V_{N},\phi_{E},\phi_{N},\phi_{U},\nabla_{x},\nabla_{y},\varepsilon_{x},\varepsilon_{y},\varepsilon_{z}\right]}^{T}$$

where *δV_E_* and *δV_N_* are the east velocity error and north velocity error, respectively, in the navigation frame; *ϕ_E_*, *ϕ_N_*, and *ϕ_U_* are the east misalignment angle, north misalignment angle, and azimuth misalignment angle, respectively, in the navigation frame; ∇*_x_* and ∇*_y_* are the right-axis and forward-axis accelerometer bias, respectively, in the body frame, *b*; and *ε_x_*, *ε_y_*, and *ε_z_* are the right-axis, forward-axis, and up-axis gyro drift, respectively, in the body frame.

The SINS’ state equation can be constructed as follows.

(11) $$\dot{X}(t)=F(t)X(t)+G(t)W(t)$$

where X(t) is the (*n* × 1) state estimate, $\dot{X}(t)$ is the (*n* × 1) one step predicted state, G(t) is the system noise matrix, and W(t) is the zero mean Gaussian white noise. F(t) is the (*n* × *n*) state transition matrix, and it can be expressed as follows:

(12) $$F(t)=\left[\begin{array}{cc} F_{1}(t) & C(t)\\ 0_{5\times 7} & 0_{5\times 5} \end{array}\right].$$

In Equation (12), $F_{1}(t)$ can be represented in concrete formulas as

(13) $$\begin{array}{l} F_{1}(t)=\\ \left[\begin{array}{ccccccc} 0 & 0 & 0 & \frac{1}{R_{M}} & 0 & 0 & 0\\ \frac{V_{E}}{R_{N}}tgL\sec L & 0 & \frac{\sec L}{R_{N}} & 0 & 0 & 0 & 0\\ 2V_{N}\omega_{ie}\cos L+\frac{V_{E}V_{N}}{R_{N}}\sec^{2}L & 0 & \frac{V_{N}}{R_{N}}tgL & 2\omega_{ie}\sin L+\frac{V_{E}}{R_{N}}tgL & 0 & -f_{U} & f_{N}\\ -(2V_{E}\omega_{ie}\cos L+\frac{V_{E}^{2}}{R_{N}}\sec^{2}L) & 0 & -2(\omega_{ie}\sin L+\frac{V_{E}}{R_{N}}tgL) & 0 & f_{U} & 0 & -f_{E}\\ 0 & 0 & 0 & -\frac{1}{R_{M}} & \omega_{ie}\sin L+\frac{V_{E}}{R_{N}}tgL & -(\omega_{ie}\cos L+\frac{V_{E}}{R_{N}}) & 0\\ -\omega_{ie}\sin L & 0 & \frac{1}{R_{N}} & 0 & -\omega_{ie}\sin L-\frac{V_{E}}{R_{N}}tgL & 0 & -\frac{V_{N}}{R_{M}}\\ \omega_{ie}\cos L+\frac{V_{E}}{R_{N}}\sec^{2}L & 0 & \frac{tgL}{R_{N}} & 0 & \omega_{ie}\cos L+\frac{V_{E}}{R_{N}} & \frac{V_{N}}{R_{M}} & 0 \end{array}\right] \end{array}$$

C(t) can be represented in concrete formulas as

(14) $$C(t)=\left[\begin{array}{ccccc} 0 & 0 & 0 & 0 & 0\\ 0 & 0 & 0 & 0 & 0\\ T_{11} & T_{12} & 0 & 0 & 0\\ T_{21} & T_{22} & 0 & 0 & 0\\ 0 & 0 & -T_{11} & -T_{12} & -T_{13}\\ 0 & 0 & -T_{21} & -T_{22} & -T_{23}\\ 0 & 0 & -T_{31} & -T_{32} & -T_{33} \end{array}\right].$$

In Equation (14), the SINS attitude matrix is defined as $C_{b}^{n}={\left[T_{ij}\right]}_{3\times 3}$.

### 2.2. Measurement Model

In the model of transfer alignment, velocity, and attitude differences between M-SINS and S-SINS are chosen as the measurements. Thus, the measurement of velocity plus attitude matching can be expressed as follows:

(15) $$Y(t)=\left[\begin{array}{l} \delta V_{E}\\ \delta V_{N}\\ \delta H\\ \delta P\\ \delta R \end{array}\right]=\left[\begin{array}{c} {\tilde{V}}_{E}^{s}-V_{E}^{m}\\ \begin{array}{l} {\tilde{V}}_{N}^{s}-V_{N}^{m}\\ {\tilde{H}}^{s}-H^{m}\\ {\tilde{P}}^{s}-P^{m}\\ {\tilde{R}}^{s}-R^{m} \end{array} \end{array}\right]$$

where Y(t) is the measurement at time *t*; *δV_E_* and *δV_N_* are the east velocity error and north velocity error, respectively; *δH*, *δP*, and *δR* are the heading angle error, pitch angle error, and roll angle error, respectively; ${\tilde{V}}_{E}^{s}$ and ${\tilde{V}}_{N}^{s}$ are the S-SINS’ east velocity and north velocity, respectively; $V_{E}^{m}$ and $V_{N}^{m}$ are the M-SINS’ east velocity and north velocity, respectively; ${\tilde{H}}^{s}$, ${\tilde{P}}^{s}$, and ${\tilde{R}}^{s}$ are the S-SINS’ heading angle, pitch angle, and roll angle, respectively; and $H^{m}$, $P^{m}$, and $R^{m}$ are the M-SINS’ heading angle, pitch angle, and roll angle, respectively.

The system’s measurement equation can be represented as follows:

(16) Y(t)=H(t)X(t)+V(t)

where *V*(*t*) is the measurement noise and *H*(*t*) is the measurement matrix. Additionally,

(17) $$H(t)=\left[\begin{array}{c} \begin{array}{cc} \;I_{2\times 2}\; & \;0_{2\times 10}\; \end{array}\\ \begin{array}{ccc} 0_{3\times 2} & \;H_{1}(t) & \;0_{3\times 7} \end{array} \end{array}\right].$$

In Equation (17), $H_{1}(t)$ can be represented in concrete formulas as

(18) $$H_{1}(t)=\left[\begin{array}{ccc} -\frac{T_{12}T_{32}}{T_{12}^{2}+T_{22}^{2}} & -\frac{T_{32}T_{22}}{T_{12}^{2}+T_{22}^{2}} & 1\\ -\frac{T_{22}}{\sqrt{1-T_{32}^{2}}} & \frac{T_{12}}{\sqrt{1-T_{32}^{2}}} & 0\\ \frac{T_{21}T_{33}-T_{31}T_{23}}{T_{31}^{2}+T_{33}^{2}} & \frac{T_{13}T_{31}-T_{11}T_{33}}{T_{31}^{2}+T_{33}^{2}} & 0 \end{array}\right].$$

### 2.3. Sensor Error Compensation Model

During the process of transfer alignment, the sensor device error has an important influence on the alignment accuracy. The sensor device error determines the SINS’ subsequent navigation performance [31]. Therefore, it is necessary to calibrate and compensate the device errors of three-axis gyros and three-axis accelerometers. The gyro drift consists of random constant drift, random slowly varying drift, and random rapidly varying drift. It can be represented as follows:

(19) $$\varepsilon_{i}^{b}=\varepsilon_{bi}^{b}+\varepsilon_{ri}^{b}+\omega_{gi}^{b}\;(i=x,y,z)$$

where $\varepsilon_{bi}^{b}$ is the random constant drift, $\varepsilon_{ri}^{b}$ is the random slowly varying drift, and $\omega_{gi}^{b}$ is the random rapidly varying drift. The value of $\varepsilon_{bi}^{b}$ depends on random factors, such as environmental conditions and electrical parameters present when the gyro starts to work. It is a random constant after every start of the gyro. The value of $\varepsilon_{ri}^{b}$ is a slowly changing quantity, and it can be described by a first-order Markov process. The value of $\omega_{gi}^{b}$ is a rapidly changing quantity, and it can be abstracted into white Gaussian noise. In the above variables, the random constant drift, $\varepsilon_{bi}^{b}$, is the main part of the gyro drift, and most gyro errors come from it. Thus, if $\varepsilon_{bi}^{b}$ can be accurately calibrated during transfer alignment, the navigation performance of the gyro will be effectively improved. In this paper, an improved adaptive compensation H∞ filtering method is used to estimate the gyro’s random constant drift, and the compensated gyro drift can be expressed as follows:

(20) $$\varepsilon_{i\_new}^{b}=\varepsilon_{bi}^{b}+\varepsilon_{ri}^{b}+\omega_{gi}^{b}-\varepsilon_{bi\_cal}^{b}\;(i=x,y,z)$$

where $\varepsilon_{i\_new}^{b}$ is the compensated gyro drift and $\varepsilon_{bi\_cal}^{b}$ is the calibrated random constant drift.

The accelerometer bias consists of random constant bias and random rapidly varying bias. It can be represented as follows:

(21) $$\nabla_{i}^{b}=\nabla_{bi}^{b}+\omega_{ai}^{b}\;(i=x,y,z)$$

where $\nabla_{bi}^{b}$ is the random constant bias and $\omega_{ai}^{b}$ is the random rapidly varying bias. The value of $\nabla_{bi}^{b}$ depends on random factors, such as environmental conditions and electrical parameters when the accelerometer starts to work. It is a random constant after every start of the accelerometer. The value of $\omega_{ai}^{b}$ is a rapidly changing quantity, and it can be regarded as white Gaussian noise. In both variables, the random constant bias $\nabla_{bi}^{b}$ is the main part of the accelerometer bias. Most accelerometer errors come from it. Here, an improved adaptive compensation H∞ filtering method is used to estimate the accelerometer’s random constant bias, and the compensated accelerometer bias can be expressed as follows:

(22) $$\nabla_{i\_new}^{b}=\nabla_{bi}^{b}+\omega_{ai}^{b}-\nabla_{bi\_cal}^{b}\;(i=x,y,z)$$

where $\nabla_{i\_new}^{b}$ is the compensated accelerometer bias and $\nabla_{bi\_cal}^{b}$ is the calibrated random constant bias.

## 3. H∞ Filtering Method

We consider the following random linear discrete-time system in Krein space [32]:

(23) $$X_{k}=\Phi_{k,k-1}X_{k-1}+\Gamma_{k-1}W_{k-1}$$

(24) $$Y_{k}=H_{k}X_{k}+V_{k}.$$

Generally, we need to estimate some arbitrary linear combination of the states, and this can be expressed as [32]:

(25) $$Z_{k}=L_{k}X_{k}$$

where *L_k_* is the (*n* × *n*) matrix and *Z_k_* is the (*n* × 1) measurement matrix. We can use *Z_k_* to expand the observed state of *Y_k_*, and the equations below are obtained:

(26) $$X_{k}=\Phi_{k,k-1}X_{k-1}+\Gamma_{k-1}W_{k-1}$$

(27) $$\left[\begin{array}{l} Y_{k}\\ Z_{k} \end{array}\right]=\left[\begin{array}{l} H_{k}\\ L_{k} \end{array}\right]X_{k}+V_{k}^{\prime }.$$

In the above equations, *W_k_* and $V_{k}^{\prime }$ are noise with bounded energy, and their statistical properties do not need to be known or assumed. *X_0_*, *W_k_*, and $V_{k}^{\prime }$ satisfy the following relationships:

(28) $$\langle \left[\begin{array}{c} X_{0}\\ W_{j}\\ V_{j}^{\prime } \end{array}\right],\left[\begin{array}{c} X_{0}\\ W_{k}\\ V_{k}^{\prime } \end{array}\right]\rangle =[\begin{array}{ccc} P_{0} & 0 & 0\\ 0 & I\delta_{jk} & 0\\ 0 & 0 & R_{\infty k}\delta_{jk} \end{array}].$$

Here,

(29) $$R_{\infty k}=\left[\begin{array}{cc} I & 0\\ 0 & -\xi^{2}I \end{array}\right].$$

In Equation (28), *δ_jk_* is the Kronecker function, i.e., $\delta_{jk}=\left\{ \begin{array}{l} 0\;(k\neq j)\\ 1\;(k=j) \end{array}\right.$. *ξ* is the robustness factor of the H∞ filter. 

Additionally,

(30) $${\hat{Z}}_{k}=F_{f}\left(Y_{0},Y_{1},\cdots ,Y_{k}\right).$$

This means that, for the estimation of *Z_k_* in the condition of the given observation value *Y_k_*, the filtering error can be defined as follows:

(31) $$e_{k}={\hat{Z}}_{k}-L_{k}X_{k}.$$

*T_k_*(*F_f_*) is defined as the transfer function from the unknown disturbance $\left\{(X_{0}-{\hat{X}}_{0}),W_{k},V_{k}\right\}$ to the filtering error {*e_k_*}, and the suboptimal H∞ filter problem can be described as follows:

**Definition** **1** (suboptimal H∞ filter problem)**.** *When given a positive number ξ *> 0*, find the suboptimal H∞ estimation to satisfy || T_k_*(*F_f_*)* || *_∞_<* ξ, that is* [3,33]

(32) $$\underset{F_{f}}{\inf}{\left\|T_{k}(F_{f})\right\|}_{\infty }^{2}=\underset{F_{f}}{\inf}\underset{X_{0},W\in h_{2},V\in h_{2}}{\sup}\frac{{\left\|e_{k}\right\|}_{2}^{2}}{{\left\|X_{0}-{\hat{X}}_{0}\right\|}_{P_{0}^{-1}}^{2}+{\left\|W_{k}\right\|}_{2}^{2}+{\left\|{V^{\prime }}_{k}\right\|}_{2}^{2}}\leq \xi^{2}.$$

*In the above equation*, *P*_0_*is a positive definite matrix. Symbol “inf” is the infimum of function, and symbol “sup” is the supremum of function.*

**Theorem** **1** (suboptimal H∞ filter problem)**.** *When given a positive numberξ* > 0, *if* (*Φ_k,k *− 1*_ Γ_k,k *− 1*_*) *is full rank, then the condition that there is a filter satisfying* || *T_k_*(*F_f_*)** || *_∞_* < *ξ* *is if and only if* [23]

(33) $$P_{k}^{-1}+H_{k}^{T}H_{k}-\xi^{-2}L_{k}^{T}L_{k}>0$$

*In the above equation*, *P_k_satisfies the recurrence Riccati equation*

(34) $$P_{k}=\Phi_{k,k-1}P_{k-1}\Phi_{k,k-1}^{T}+\Gamma_{k,k-1}\Gamma_{k,k-1}^{T}-\Phi_{k,k-1}P_{k-1}\left[\begin{array}{cc} H_{k}^{T} & L_{k}^{T} \end{array}\right]R_{e,k}^{-1}\left[\begin{array}{c} H_{k}\\ L_{k} \end{array}\right]P_{k-1}\Phi_{k,k-1}^{T}.$$

*In Equation (34)*,

(35) $$R_{e,k}=\left[\begin{array}{cc} I & 0\\ 0 & -\xi^{2}I \end{array}\right]+\left[\begin{array}{c} H_{k}\\ L_{k} \end{array}\right]P_{k-1}\left[\begin{array}{cc} H_{k}^{T} & L_{k}^{T} \end{array}\right].$$

*Derived from this, the recursive equations of the H∞ filter can be expressed as follows* [20]:

(36) $${\hat{X}}_{k+1,k}=\Phi_{k+1,k}{\hat{X}}_{k}$$

(37) $$K_{S,k+1}=P_{k}H_{k}^{T}{\left(I+H_{k}P_{k}H_{k}^{T}\right)}^{-1}$$

(38) $${ϒ}_{k+1}=\left(I-K_{S,k+1}H_{k}\right)P_{k}$$

(39) $$U_{k+1}={ϒ}_{k+1}L_{k}^{T}{\left(-\xi^{2}I+L_{k}{ϒ}_{k+1}L_{k}^{T}\right)}^{-1}L_{k}$$

(40) $$P_{k+1}=\Phi_{k+1,k}P_{k}\Phi_{k+1,k}^{T}-\Phi_{k+1,k}\left[\left(I-U_{k+1}\right)K_{S,k+1}H_{k+1}+U_{k+1}\right]P_{k}\Phi_{k+1,k}^{T}+\Gamma_{k}\Gamma_{k}^{T}$$

(41) $$K_{k+1}=P_{k+1}H_{k+1}^{T}{\left(H_{k+1}P_{k+1}H_{k+1}^{T}+I\right)}^{-1}$$

(42) $${\hat{X}}_{k+1}={\hat{X}}_{k+1,k}+K_{k+1}\left(Y_{k+1}-H_{k+1}{\hat{X}}_{k+1,k}\right).$$

## 4. The Proposed Adaptive Compensation H∞ Filtering Method

**Figure 1:**
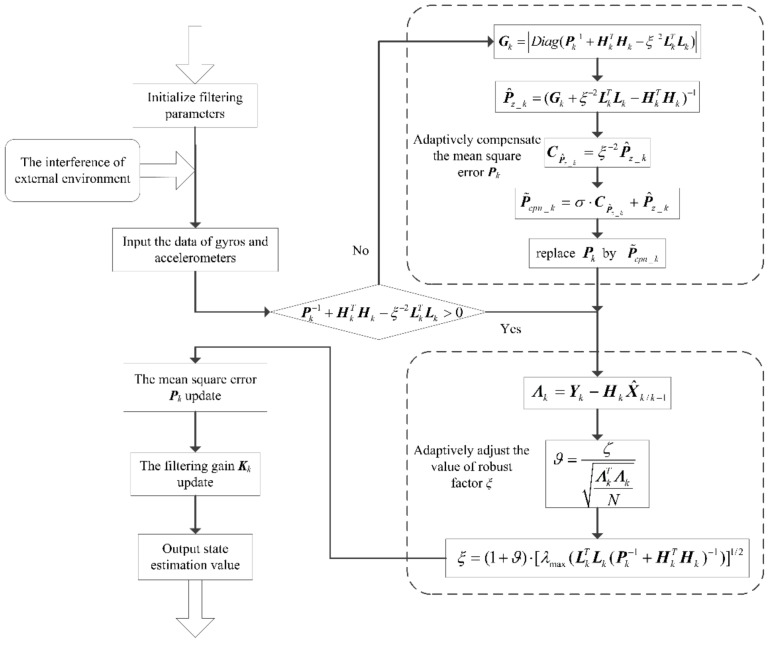
Block diagram of the proposed adaptive compensation H∞ filtering method.

In the robust H∞ filtering method, Equation (33) may not be satisfied completely due to the system itself or the uncertainty of the model. This inevitably leads to no convergence or a slow convergence rate of the filter, and the requirement of system performance is unable to be met [25]. To solve the problem and improve the estimation accuracy of filtering under a complex dynamic environment, this paper proposes an adaptive compensation H∞ filtering method. The block diagram of the proposed adaptive compensation H∞ filtering method is shown in Figure 1. The adaptive filtering process mainly consists of adaptively compensating the mean square error *P_k_* and adaptively adjusting the value of robust factor *ξ*.

Firstly, the composition of Equation (33) needs to be analyzed. Equation (33) can be rewritten as follows:

(43) $$P_{k}^{-1}+H_{k}^{T}H_{k}-\xi^{-2}L_{k}^{T}L_{k}>0.$$

The left side of Equation (43) consists of three parts: the first part ($P_{k}^{-1}$), the second part ($H_{k}^{T}H_{k}$), and the third part ($\xi^{-2}L_{k}^{T}L_{k}$). In $P_{k}^{-1}$, because *P_k_* can update by Equation (40) and the initial *P_k_* is a given positive definite matrix that meets the requirement of Equation (43), $P_{k}^{-1}$ is a matrix that can be adjusted. In $H_{k}^{T}H_{k}$, because *H_k_* comes from the observation of Equation (24), it is a matrix that cannot be adjusted when the system model has been determined. Since the symbol is negative in $\xi^{-2}L_{k}^{T}L_{k}$, this part is the most common reason why the requirement of Equation (43) cannot be met. The *L_k_* in $\xi^{-2}L_{k}^{T}L_{k}$ comes from Equation (25), and it is a predetermined matrix that cannot be adjusted. However, the robustness factor, *ξ*, is a parameter that can be adjusted dynamically, so the value of *ξ* can increase to reduce the influence of the third part, $\xi^{-2}L_{k}^{T}L_{k}$. However, the value of *ξ* cannot increase unboundedly in practical engineering applications. If the value of *ξ* becomes too big, the fluctuation range of the filtering error curve will increase. Therefore, the system state estimation will be affected. Through the above analysis, the only way to remedy this is to adjust *P_k_* dynamically to meet the convergence condition of Equation (43).

In this paper, an adaptive compensation H∞ filtering method is proposed to keep the left side of Equation (43) positive. Firstly, the positive definiteness of $P_{k}^{-1}+H_{k}^{T}H_{k}-\xi^{-2}L_{k}^{T}L_{k}$ is judged. If $P_{k}^{-1}+H_{k}^{T}H_{k}-\xi^{-2}L_{k}^{T}L_{k}$ is not positive, then the absolute value of the diagonal elements of the matrix $P_{k}^{-1}+H_{k}^{T}H_{k}-\xi^{-2}L_{k}^{T}L_{k}$ is retained, and the rest of the elements are all set to zero. The modified matrix $G_{k}$ can be expressed as follows:

(44) $$G_{k}=\left|Diag(P_{k}^{-1}+H_{k}^{T}H_{k}-\xi^{-2}L_{k}^{T}L_{k})\right|$$

where the symbol “| |”denotes taking the absolute value of the element.

Additionally,

(45) $${\hat{P}}_{z\_k}^{-1}+H_{k}^{T}H_{k}-\xi^{-2}L_{k}^{T}L_{k}=G_{k}$$

where ${\hat{P}}_{z\_k}^{}$ is a positive matrix that meets the requirement of Equation (43). Using the transformation of Equation (45), ${\hat{P}}_{z\_k}^{}$ can be expressed as follows:

(46) $${\hat{P}}_{z\_k}={\left(G_{k}+\xi^{-2}L_{k}^{T}L_{k}-H_{k}^{T}H_{k}\right)}^{-1}.$$

In order to further keep the left side of Equation (43) positive, the recursive matrix ${\hat{P}}_{z\_k}^{}$ needs to be compensated. The compensated recursive matrix ${\hat{P}}_{z\_k}^{}$ can be written as follows:

(47) $${\tilde{P}}_{cpn\_k}=\sigma \cdot C_{{\hat{P}}_{z\_k}}+{\hat{P}}_{z\_k}$$

where ${\tilde{P}}_{cpn\_k}$ is the mean square error matrix that has been compensated; *σ* is the gain coefficient that can be determined according to the practical system; and $C_{{\hat{P}}_{z\_k}}$ is an adaptive compensation matrix. In order to make full use of the robustness factor the concrete form of the adaptive compensation matrix $C_{{\hat{P}}_{z\_k}}$ can be written as follows.

(48) $$C_{{\hat{P}}_{z\_k}}=\xi^{-2}{\hat{P}}_{z\_k}.$$

In Equation (48), the robustness factor controls the degree of compensation. When the value of the robustness factor is small, it indicates that the nonpositive definite situation on the left side of Equation (43) caused by $\xi^{-2}L_{k}^{T}L_{k}$ is obvious, and the ${\hat{P}}_{z\_k}^{}$ should be compensated largely. When the value of the robustness factor is big, it indicates that the nonpositive definite situation on the left side of Equation (43) caused by $\xi^{-2}L_{k}^{T}L_{k}$ is not obvious, and the ${\hat{P}}_{z\_k}^{}$ should not have to make too much compensation.

Based on Equations (44)–(48), the modified mean square error matrix ${\tilde{P}}_{cpn\_k}$ can be obtained, and it is substituted into the adaptive compensation H∞ filter to estimate the variables of the system state equation.

In the adaptive compensation H∞ filtering method, the robustness factor plays an important role in filtering accuracy and robustness [20]. When the value of the robustness factor is too small, it means that the system has good robustness, but the filtering accuracy of the system becomes low. When the value of the robustness factor is too big, it means that the system has bad stability and even leads to divergence. In practical engineering applications, the value of the robustness factor needs to be debugged repeatedly before applying a suitable value to use, and the value of the robustness factor is often empirically determined. This will waste a lot of time, and the value of *ξ* is not guaranteed to be optimal. Moreover, as the robustness factor is fixed during the process of transfer alignment, the filtering estimation error will increase when there are large disturbances at certain times. Therefore, it cannot achieve the optimal balance between filtering accuracy and robustness when the external environment changes dynamically. To solve this problem, an adaptive optimization method of the robustness factor is proposed to improve the performance of the adaptive compensation H∞ filter.

In the process of transfer alignment, the filter innovation ($\Lambda_{k}$) can reflect the degree of convergence and divergence of the filter. $\Lambda_{k}$ can be calculated as follows:

(49) $$\Lambda_{k}=Y_{k}-H_{k}{\hat{X}}_{k/k-1}.$$

From Equation (49), the prediction error can be described by the innovation sequence’s variance, $E\;[\Lambda_{k}^{T}\Lambda_{k}]$. Under ideal conditions, the filter innovation $\Lambda_{k}$ is a zero mean Gaussian white noise sequence. However, in real systems, both the changes of the system model and the anomaly of observed states can cause a change in the statistical properties of the innovation sequence. When the value of $\Lambda_{k}^{T}\Lambda_{k}$ is too big, it indicates that there is a large disturbance in the transfer alignment system, and the filter has the possibility of undergoing divergence. Hence, the robustness of the adaptive compensation H∞ filter is of great importance at this point, and the value of the robustness factor should be reduced to compensate the large error that is caused by $\Lambda_{k}^{T}\Lambda_{k}$. When the value of $\Lambda_{k}^{T}\Lambda_{k}$ is too small, it indicates that there is little disturbance in the transfer alignment system, so the value of the robustness factor should be increased to improve the filtering accuracy. In the whole process of transfer alignment, the robustness factor of the adaptive compensation H∞ filter varies inversely to $\Lambda_{k}^{T}\Lambda_{k}$.

**Theorem** **2.** *Assume that M and N are two n-order Hermite matrices, M *> 0*, and N *≥ 0*. Then, M *>* N is equal to*$\lambda_{\max}(NM^{-1})\;<\;1$ [20]. *Here, λ_max_(M) represents the maximum eigenvalue of matrix M*.

According to Theorem 2, the conditional Equation (33) of the H∞ filter can be transformed into the following form:

(50) $$\xi^{2}>\lambda_{\max}(L_{k}^{T}L_{k}{\left(P_{k}^{-1}+H_{k}^{T}H_{k}\right)}^{-1}).$$

Additionally, the value of the robustness factor can be expressed as follows:

(51) $$\xi =(1+\vartheta )\cdot {\left[\lambda_{\max}(L_{k}^{T}L_{k}{\left(P_{k}^{-1}+H_{k}^{T}H_{k}\right)}^{-1})\right]}^{1/2}.$$

In Equation (51), the coefficient *ϑ* > 0. As the value of the robustness factor is inversely proportional to $\Lambda_{k}^{T}\Lambda_{k}$, we can make *ϑ* and $\Lambda_{k}^{T}\Lambda_{k}$ satisfy the following relationship:

(52) $$\vartheta =\frac{\zeta }{\sqrt{\frac{\Lambda_{k}^{T}\Lambda_{k}}{N}}}$$

where *ζ* is correlation coefficient and *ζ* > 0. The value of *ζ* can be determined by the experiment of the real system. *N* is the dimension of the filtering states. For a specific real system, once the values of *ζ* and *N* are determined, respectively, they will not change any more. Therefore, the value of coefficient *ϑ* is only related to filter innovation during the process of transfer alignment. When filter innovation ($\Lambda_{k}$) increases, the value of coefficient *ϑ* decreases, so the robustness factor of the adaptive compensation H∞ filter will be decreased to improve the robustness of the transfer alignment system. On the contrary, when filter innovation $\Lambda_{k}$ decreases, the value of coefficient *ϑ* increases. Then, the robustness factor of the adaptive compensation H∞ filter will be increased to improve the estimation accuracy of the transfer alignment system. Consequently, the relationship between the robustness factor of adaptive compensation H∞ filter and $\Lambda_{k}^{T}\Lambda_{k}$ can be established, and the value of the robustness factor will change dynamically when $\Lambda_{k}$ changes.

**Figure 2:**
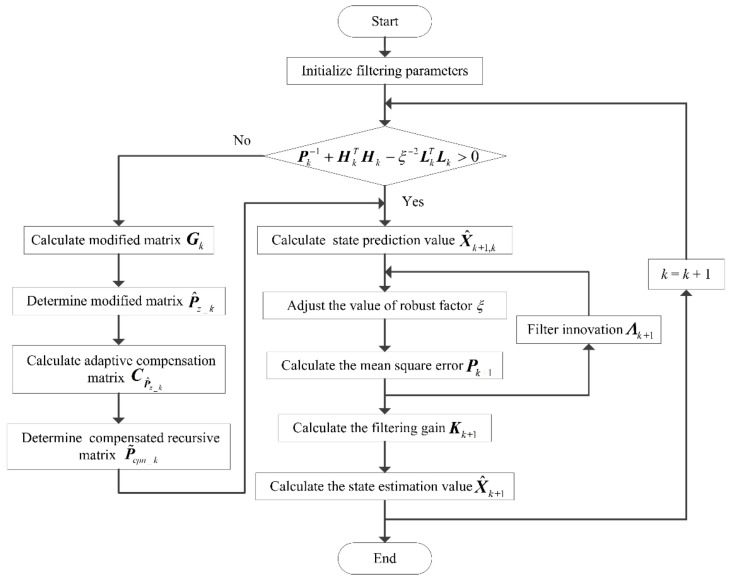
Flow chart of the proposed adaptive compensation H∞ filtering method.

By comparing the adaptive compensation H∞ filter and the H∞ filter, it can be seen that the robustness factor of the adaptive compensation H∞ filter is dynamically adjusted according to the value of $\Lambda_{k}$. Unlike the H∞ filter, the adaptive compensation H∞ filter can adjust the robustness factor adaptively when the real system environment changes. Therefore, the filtering estimation accuracy and robustness of the transfer alignment system can be balanced dynamically by using the proposed adaptive compensation H∞ filtering method. The flow chart of the proposed adaptive compensation H∞ filtering method is shown in Figure 2.

## 5. Experimental Results and Discussion

### 5.1. Experimental Settings

**Figure 3:**
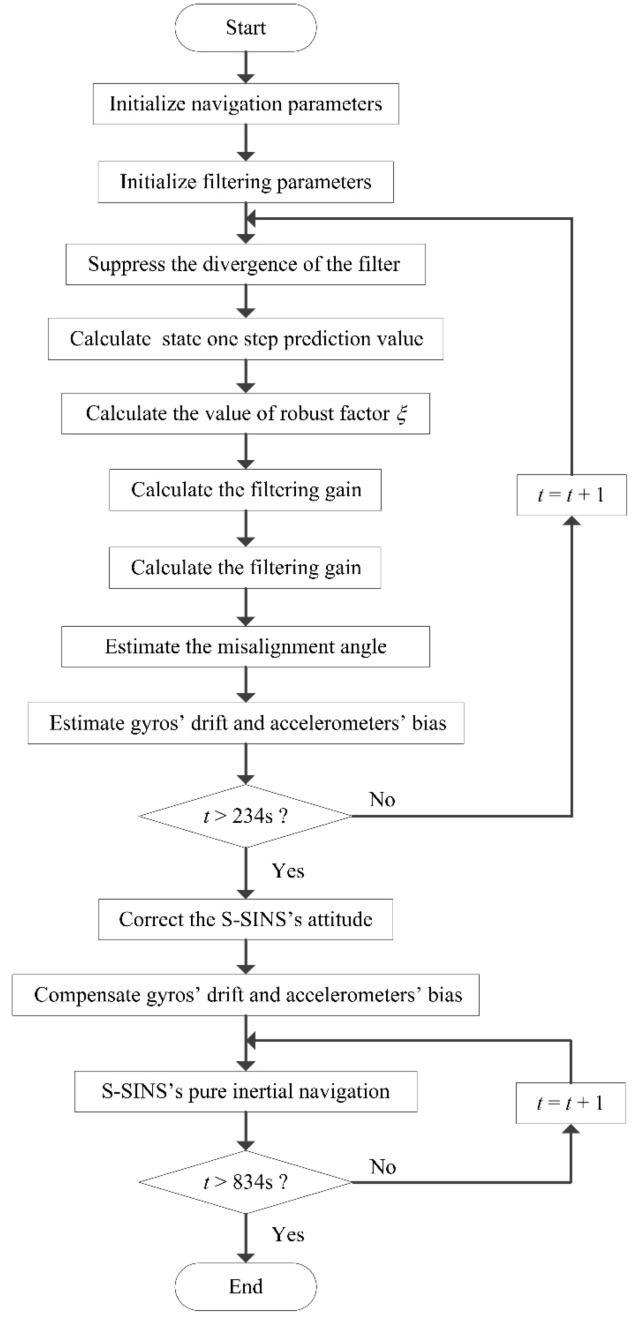
Flow chart of aerial transfer alignment based on the adaptive compensation. H∞ filtering method. S-SINS is slave SINS (strapdown inertial navigation system).

With the purpose of verifying the proposed new adaptive compensation H∞ filtering method, an aerial transfer alignment experiment in a real system was conducted. The aerial transfer alignment experiment was conducted for the airborne distributed position and orientation system (POS), which depends on transfer alignment to obtain high accuracy motion parameters of S-SINSs by using accurate motion information of M-SINS, including position, velocity and attitude. Furthermore, the aerial transfer alignment experiment was conducted in the Y-7 transport aircraft. The real aerial transfer alignment experiment was carried out in Nanjing in an open area. The specific location information of this experiment was 32° N and 119° E. The transfer alignment system contained the M-SINS, S-SINS, and navigation computer, which were connected by communication lines. The positions of both M-SINS and S-SINS were kept strictly fixed in the experimental aircraft. The update frequencies for M-SINS and S-SINS were all 200 Hz. The strapdown algorithm update cycle was 5 ms. The three-axis gyro random constant drifts of M-SINS were 0.02°/h. The three-axis accelerometer random constant biases of M-SINS were 0.05 mg. The three-axis gyro random constant drifts of S-SINS were 1.2°/h. The three-axis accelerometer random constant biases of S-SINS were 0.1 mg. The reference baseline information was provided by M-SINS during the process of aerial transfer alignment, and the sensor parameters of M-SINS were far better than those of S-SINS. The flow chart of the aerial transfer alignment experiment based on the proposed adaptive compensation H∞ filtering method is presented in Figure 3.

**Figure 4:**
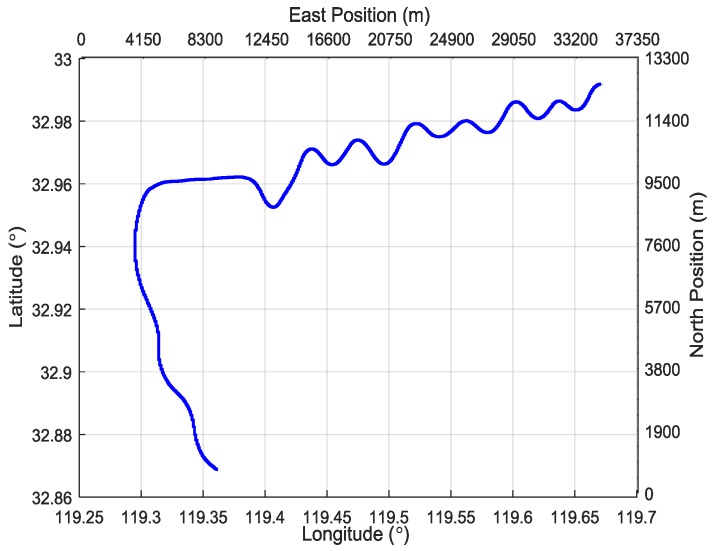
The trajectory of the aerial transfer alignment experiment.

**Figure 5:**
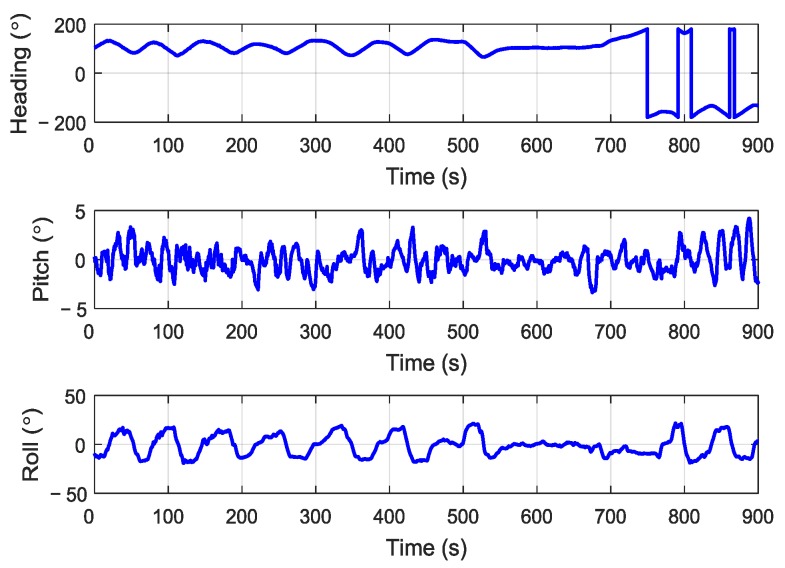
The attitude curves of master SINS (M-SINS).

Firstly, the system took 234 s to conduct the aerial transfer alignment on a moving base. Then the S-SINS of the aerial transfer alignment system began with 600 s of pure inertial navigation. Throughout the whole process, the navigation computer recorded the output data of M-SINS and S-SINS, which were transmitted through the communication lines in real time. The movement trajectory of the aerial transfer alignment experiment is shown in Figure 4. For the period of aerial transfer alignment and pure inertial navigation, the M-SINS’ attitude curves of heading, pitch, and roll are shown in Figure 5. The M-SINS’ heading fluctuated between 200° and −200° after 740 s, and the M-SINS’ attitudes had large fluctuations during the whole process.

**Figure 6:**
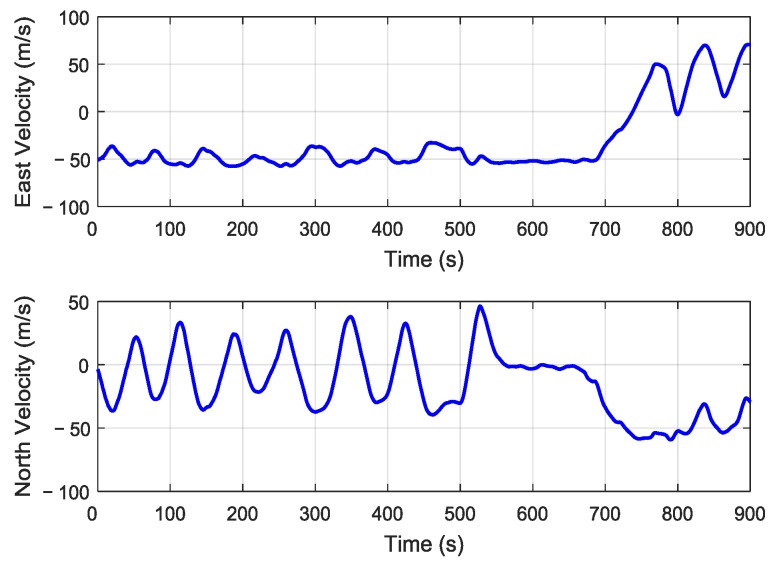
The east velocity and north velocity of M-SINS.

The M-SINS’ curves of east velocity and north velocity are shown in Figure 6. Before 700 s, the east velocity was approximately −50 m/s, then the velocity increased to 50 m/s. The north velocity fluctuated between 50 m/s and −50 m/s before 700 s, then the velocity decreased to −50 m/s. It can be seen that the aircraft had a high-flying speed throughout the whole transfer alignment experiment. 

### 5.2. Experimental Results and Discussion

**Figure 7:**
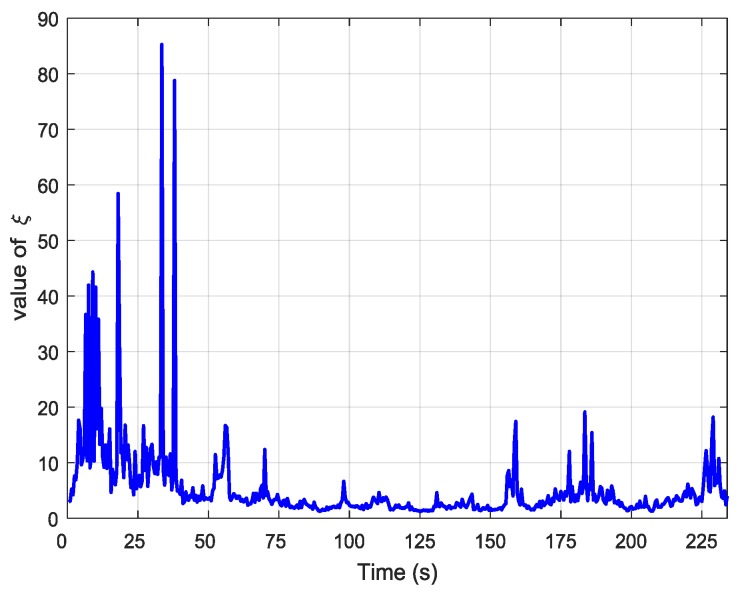
The value of the robustness factor in the adaptive. compensation H∞ filter.

In the proposed adaptive compensation H∞ filtering method, the robustness factor was adjusted dynamically according to the change in the external environment. If the system had large external disturbances during the transfer alignment process, the value of the robustness factor decreased to improve the robustness of the system. If the system had small external disturbances when conducting transfer alignment, the value of the robustness factor increased to improve the estimation accuracy of the system states. Figure 7 shows the value of the robustness factor in the adaptive compensation H∞ filtering method during the aerial transfer alignment experiment.

**Figure 8:**
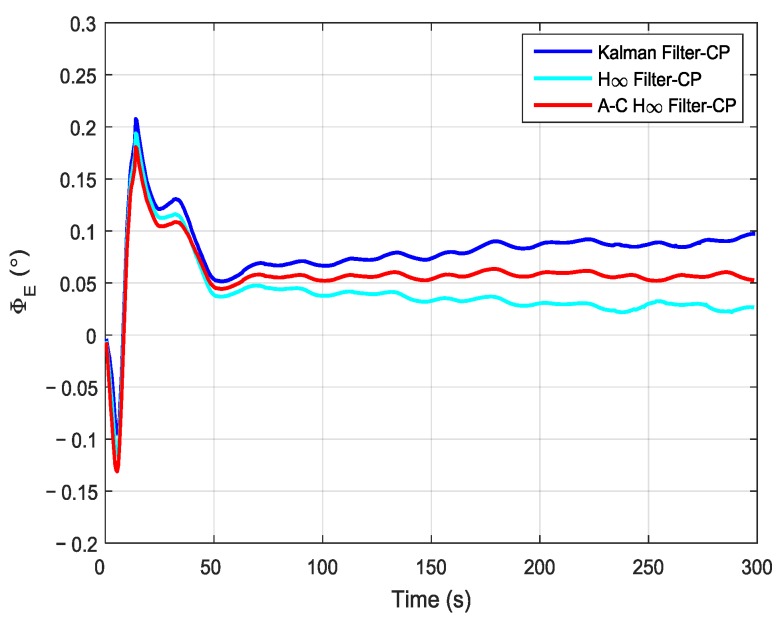
The estimation curves of the east misalignment angle. A–C denotes “adaptive compensation”; CP denotes “compensate gyro’s drift and accelerometer’s bias”.

**Figure 9:**
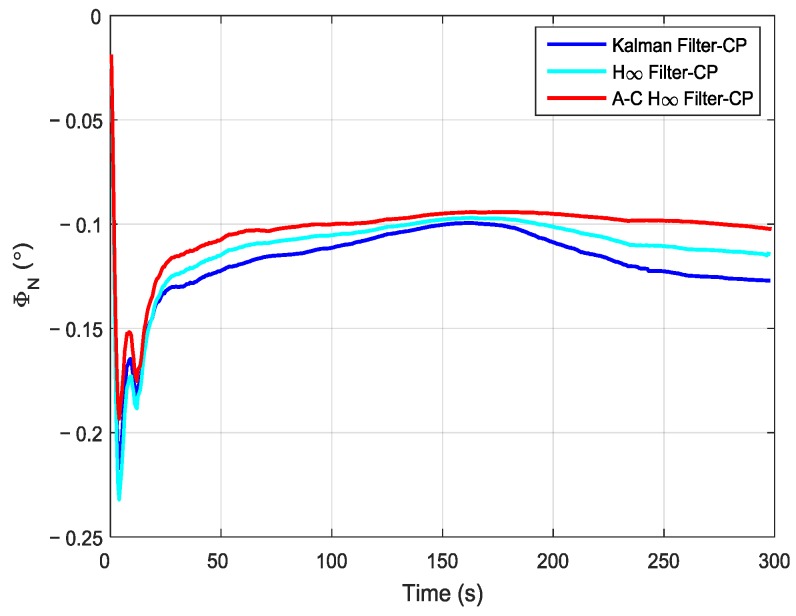
The estimation curves of the north misalignment angle.

**Figure 10:**
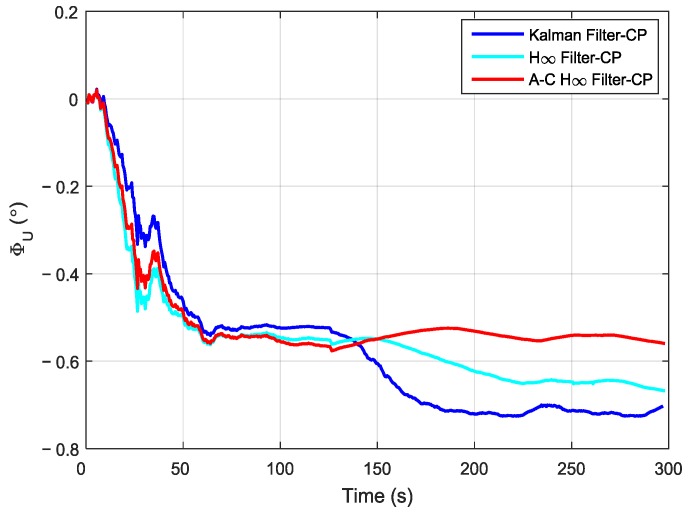
The estimation curves of the azimuth misalignment angle.

In order to compare the performance of different filtering methods, the Kalman filter, the H∞ filter, and the adaptive compensation H∞ filter were used during the process of the aerial transfer alignment experiment. The robust factor *ξ* of the H∞ filter was set as 1.89, which is the optimal robust factor of H∞ filter in this aerial transfer alignment experiment. The estimation curves of the east misalignment angle, north misalignment angle, and azimuth misalignment angle using the mentioned filtering methods are presented in Figure 8, Figure 9 and Figure 10.

In the figures, the blue lines represent the estimation curves of the Kalman filter, the cyan lines represent the estimation curves of the H∞ filter, and the red lines represent the estimation curves of the adaptive compensation H∞ filter. As shown in Figure 8, the estimation curves of the east misalignment angle (*ϕ_E_*) were approximately 0.05°. By using the Kalman filter method, the estimation curve of *ϕ_E_* increased slowly, and it reached approximately 0.1° after 225 s. When using the H∞ filter method, the estimation curve of *ϕ_E_* decreased slowly. It reached approximately 0.025° after 225 s. In contrast, when using the adaptive compensation H∞ filtering method, the estimation curve of *ϕ_E_* stabilized around 0.05°, and the estimation curve converged and tended to be stable rapidly. Furthermore, the estimation curve is more stable than those using the Kalman filtering method and the H∞ filtering method. This can be used as an important reference standard to measure whether the accuracy is good or not when conducting the aerial transfer alignment experiment. Therefore, it can be verified that the estimated value was more accurate than those found using the other two methods. 

It can be seen from Figure 9 that, when using the Kalman filtering method, the estimation curve of the north misalignment angle (*ϕ_N_*) was approximately −0.1° after 150 s. However, after that, the estimation value began to decrease rapidly, and it was approximately −0.12° after 234 s. When using the H∞ filtering method, the estimation curve of *ϕ_N_* was near −0.1° after 150 s, and the estimation value decreased more slowly than when the Kalman filtering method was used. In contrast, by using the adaptive compensation H∞ filtering method, the estimation curve of *ϕ_N_* reached −0.1° after 100 s, and it maintained a stable state. It can be seen that the estimation value by using the adaptive compensation H∞ filtering method was more stable compared with that of the Kalman filtering method and the H∞ filtering method, and it was more beneficial to improving the accuracy of S-SINS’ transfer alignment.

In Figure 10, the estimation curves of the azimuth misalignment angle (*ϕ_U_*) found using the three methods are presented. The estimation curve of *ϕ_U_* by using the Kalman filter was approximately −0.55° after 70 s. However, the estimation value began to decrease rapidly after 125 s, and the estimation curve had large fluctuations. When using the H∞ filtering method, the estimation curve of *ϕ_U_* began to decrease after 150 s. However, the fluctuations of the estimation curve using the H∞ filtering method were smaller than those using the Kalman filtering method. When using the adaptive compensation H∞ filtering method, the estimation curve of *ϕ_U_* was approximately −0.55° after 70 s. The estimation value became stable. The fluctuations of the estimation curve using the adaptive compensation H∞ filtering method were the smallest amongst those of the three methods. Therefore, these can verify the superiority of the adaptive compensation H∞ filtering method.

**Figure 11:**
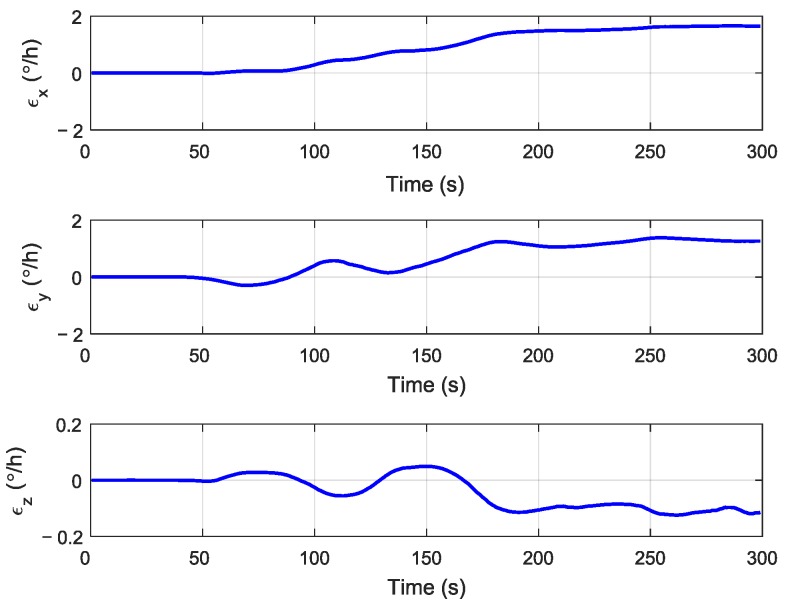
The estimation curves of the gyros’ random constant drift during the aerial transfer alignment experiment.

During the aerial transfer alignment experiment, the gyro’s random constant drift and the accelerometer’s random constant bias could be calibrated online by using the adaptive compensation H∞ filtering method. The gyro drift and the accelerometer bias were compensated effectively when the transfer alignment was completed. Figure 11 shows the estimation curves of the three-axis gyros’ random constant drift during the aerial transfer alignment experiment. The estimation curve of the *x*-axis gyros’ random constant drift gradually converged during the process of transfer alignment, and the estimation value stabilized at approximately 1.5°/h after 175 s. The estimation curve of the *y*-axis gyros’ random constant drift had small fluctuations, and the estimation value stabilized at approximately 1.2°/h after 175 s. The estimation curve of the *z*-axis gyros’ random constant drift had some fluctuations, and, after 190 s, the estimation value gradually stabilized at approximately −0.1°/h.

**Figure 12:**
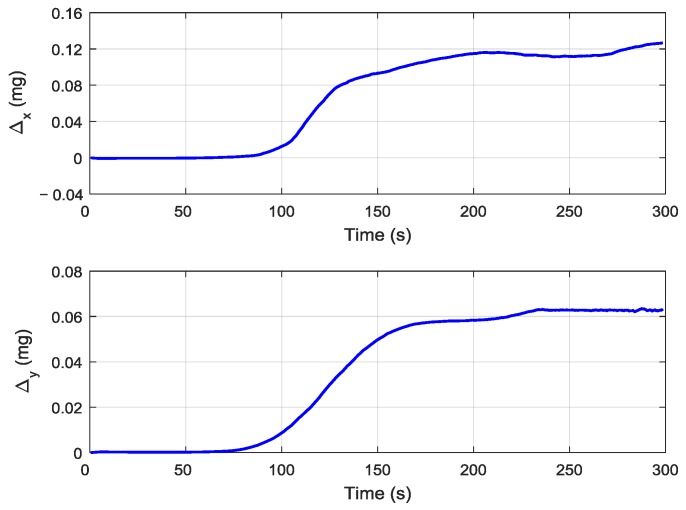
The estimation curves of the accelerometer’s random constant bias during the aerial transfer alignment experiment.

Figure 12 shows the estimation curves of the *x*-axis and *y*-axis accelerometers’ random constant bias by using the adaptive compensation H∞ filtering method in the aerial transfer alignment experiment. It can be seen that the estimation curve of the *x*-axis accelerometer’s random constant bias gradually converged, and the estimation value stabilized at approximately 0.12 mg after 200 s. The estimation curve of the *y*-axis accelerometer’s random constant bias had small fluctuations and the estimation value was approximately 0.06 mg after 200 s. Therefore, the *x*-axis and *y*-axis accelerometers’ random constant bias could be calibrated effectively during the process of transfer alignment.

**Figure 13:**
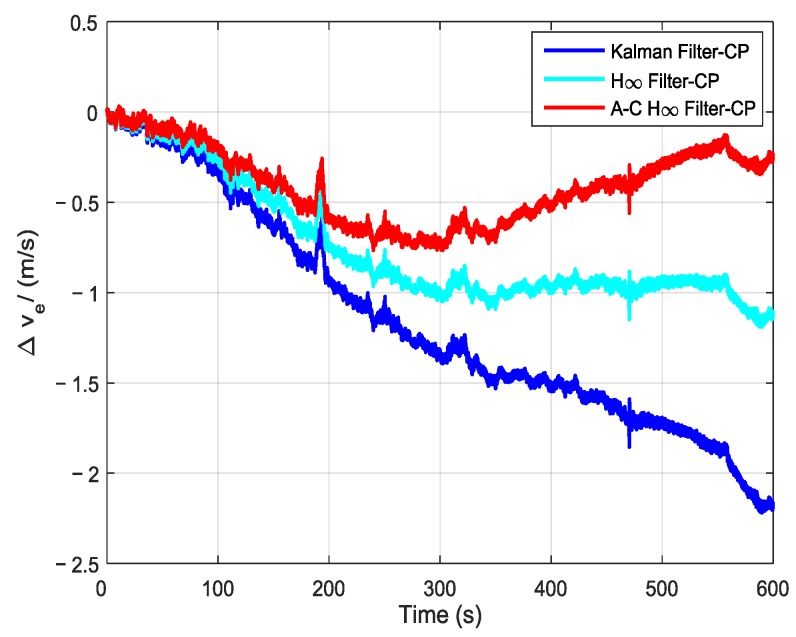
The curves of east velocity error in pure inertial navigation.

**Figure 14:**
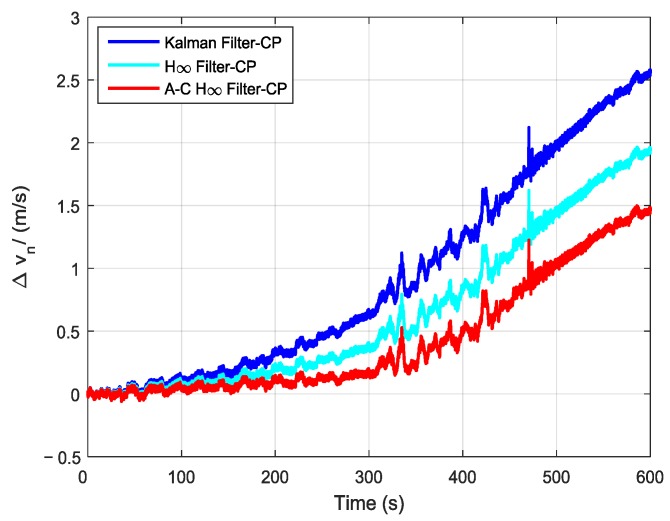
The curves of north velocity error in pure inertial navigation.

**Figure 15:**
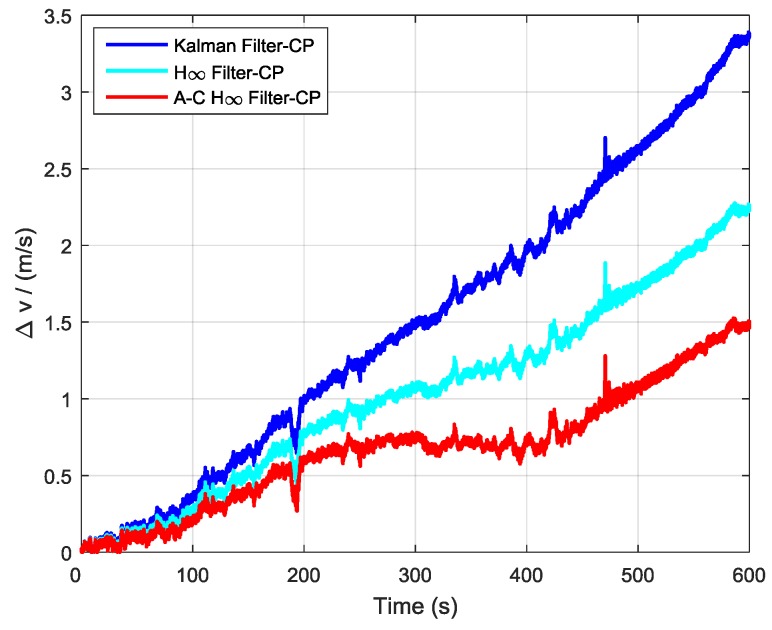
The curves of velocity error in pure inertial navigation.

In order to further verify the transfer alignment accuracy of the Kalman filtering method, the H∞ filtering method, and the proposed adaptive compensation H∞ filtering method, a pure inertial navigation experiment was conducted after the aerial transfer alignment. Since the pure inertial navigation experiment was conducted immediately after the aerial transfer alignment was completed, the pure inertial navigation accuracy had a positive correlation with the transfer alignment accuracy. The pure inertial navigation experiment lasted 10 min, and the curves of the east velocity error, north velocity error, and velocity error in the pure inertial navigation are presented in Figure 13, Figure 14 and Figure 15, respectively. The east velocity error, north velocity error, and velocity error after 10 minutes’ pure inertial navigation are listed in Table 1.

**Figure 16:**
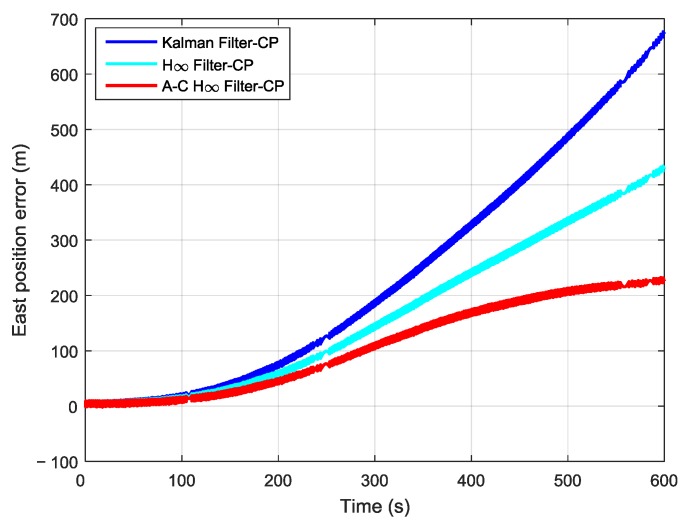
The curves of east position error in pure inertial navigation.

**Figure 17:**
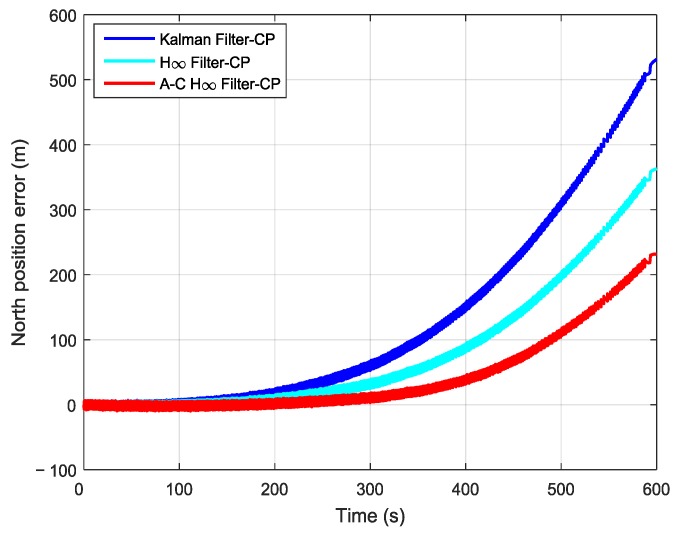
The curves of north position error in pure inertial navigation.

**Figure 18:**
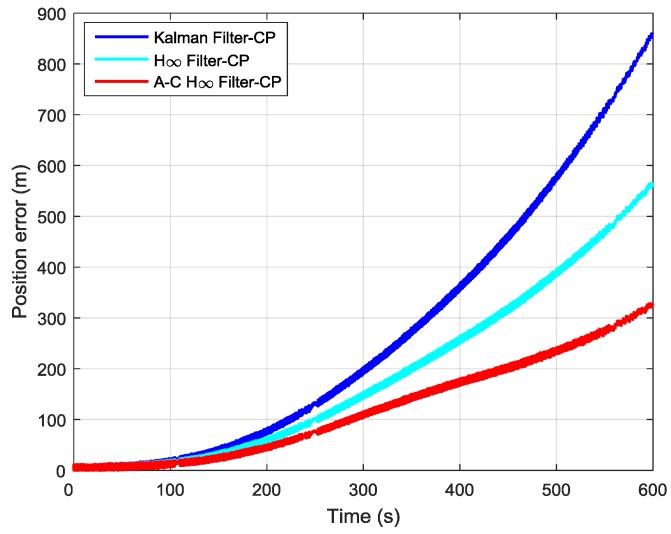
The curves of position error in pure inertial navigation.

Since the aerial transfer alignment experiment was conducted in a real system with a complex dynamic environment, the curves of east velocity error, north velocity error, and velocity error all have some irregular fluctuation phenomenon. It can be seen from Figure 13 and Table 1 that after 10 minutes’ pure inertial navigation, the east velocity error found using the Kalman filter was −2.17 m/s, the east velocity error found using H∞ filter was −1.11 m/s, and the east velocity error found using the adaptive compensation H∞ filter was −0.24 m/s. Therefore, the east velocity error found using the adaptive compensation H∞ filter was smaller than those found from using the other two methods. From Figure 14 and Table 1, the north velocity error found using the Kalman filter was 2.58 m/s, the north velocity error found using the H∞ filter was 1.93 m/s, and the north velocity error found using the adaptive compensation H∞ filter was 1.46 m/s. The north velocity error found using the adaptive compensation H∞ filter was reduced by 43.41% and 24.35%, respectively, compared to the values found by using the Kalman filter and the H∞ filter. From Figure 15 and Table 1, after 10 minutes of pure inertial navigation, the velocity error found using the Kalman filter was 3.38 m/s, the velocity error found using the H∞ filter was 2.26 m/s, and the velocity error found using the adaptive compensation H∞ filter was 1.51 m/s. The velocity error found by using the adaptive compensation H∞ filter was reduced by 55.33% and 33.19%, respectively, compared to the values found by using the Kalman filter and the H∞ filter. Based on the pure inertial navigation experiment, the curves of the east position error, north position error, and position error are presented in Figure 16, Figure 17 and Figure 18, respectively. The east position error, north position error, and position error after 10 minutes’ pure inertial navigation are listed in Table 2.

It can be seen from Figure 16 and Table 2 that after 10 min of pure inertial navigation, the east position error found using the Kalman filter was 673.12 m, the east position error found using the H∞ filter was 433.85 m, and the east position error found using the adaptive compensation H∞ filter was 226.07 m. Thus, the east position error found using the adaptive compensation H∞ filter was reduced dramatically compared with those found using the other two methods. From Figure 17 and Table 2, the north position error found using the Kalman filter was 531.41 m, the north position error found using the H∞ filter was 362.93 m, and the north position error found using the adaptive compensation H∞ filter was 231.56 m. The north position error found using the adaptive compensation H∞ filter was reduced by 56.43% and 36.20%, respectively, compared to the values found by using the Kalman filter and the H∞ filter. It is indicated from Figure 18 and Table 2 that after 10 minutes of pure inertial navigation, the position error found using Kalman filter was 857.62 m, the position error found using the H∞ filter was 565.38 m, and the position error found using the adaptive compensation H∞ filter was 323.69 m. As a result, the position error found using the adaptive compensation H∞ filter was reduced by 62.26% and 42.75%, respectively, compared to the values found using the Kalman filter and the H∞ filter. Since the pure inertial navigation accuracy has a positive correlation with the aerial transfer alignment accuracy, the smaller the position error is, the higher the transfer alignment accuracy is. In consequence, it can be verified that the aerial transfer alignment accuracy of the adaptive compensation H∞ filter is the highest among the three methods. 

**Figure 19:**
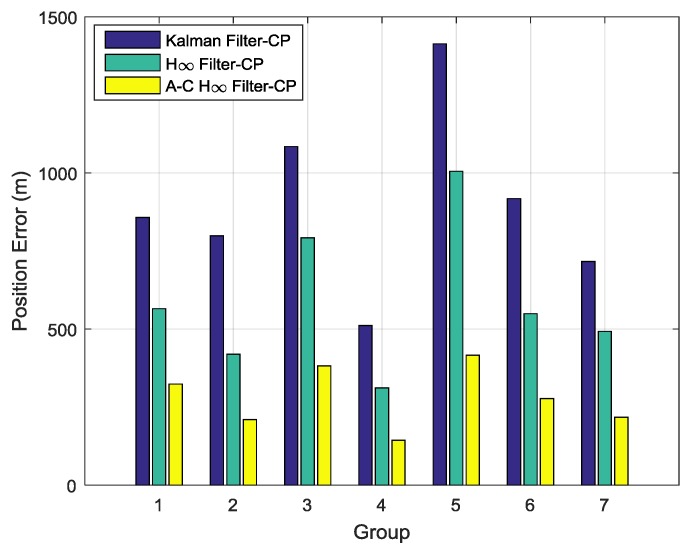
The histograms of position errors in seven groups of aerial experiment.

In order to further compare the above three filtering methods when conducting transfer alignment, a total of seven groups of aerial transfer alignment experiments were designed. The moving trajectories in the seven groups of aerial transfer experiments were different from each other and the trajectories of the aircraft were random in the whole process of experiments. The changes of flying speed and heading, pitch, and roll of M-SINS in the seven groups of aerial experiments were also different from each other. After 234 s of transfer alignment, the S-SINS started 10 min of pure inertial navigation in all groups of aerial experiments. The histograms of position errors after 10 min of pure inertial navigation in seven groups of aerial experiment are shown in Figure 19. The position errors in the seven groups of aerial experiments are listed in Table 3. 

From Figure 19 and Table 3, the position errors by using the Kalman filter, the H∞ filter, and the adaptive compensation H∞ filter are compared to each other. It can be seen that the position errors by using the Kalman filter were the largest in the seven groups of aerial experiments. Furthermore, all the position errors by using the H∞ filter were smaller than the position errors by using the Kalman filter, but they were larger than the position errors by using the adaptive compensation H∞ filter. In other words, all the position errors by using the adaptive compensation H∞ filter were the smallest in the seven groups of aerial experiments. Therefore, it can be indicated that the transfer alignment accuracy by using the adaptive compensation H∞ filter was higher than the other two methods, which fully demonstrated the advantages of the proposed method.

## 6. Conclusions

In this paper, an adaptive compensation H∞ filtering method for transfer alignment under a complex dynamic environment was presented. A transfer alignment model containing the SINS error dynamics model and the velocity plus attitude matching measurement model was constructed. With the purpose of improving the accuracy of inertial sensors, error compensation models, which can calibrate and compensate gyros’ random constant drift and accelerometer’s random constant bias online, were established. In order to effectively suppress the filtering divergence when conducting transfer alignment, the cause of filtering divergence was analyzed in detail and the mean square error matrix was dynamically modified and optimized to satisfy the convergence condition in the adaptive compensation H∞ filter. Furthermore, to improve the accuracy of the transfer alignment system, the robustness factor of adaptive compensation H∞ filter was dynamically adjusted with the change of the complex external environment. 

In order to verify the effectiveness of the adaptive compensation H∞ filtering method when conducting transfer alignment, a total of seven groups of aerial transfer alignment experiments were performed. It can be seen from the experimental results that when using the adaptive compensation H∞ filtering method, the estimation values of *ϕ_E_*, *ϕ_N_*, *ϕ_U_* were more stable than those using the Kalman filtering method and the H∞ filtering method, and the estimation curves converged and tended to stablize rapidly. This is an important reference standard to verify that the estimated value by using the adaptive compensation H∞ filtering method was more accurate than those found using the other two methods. In order to further compare the transfer alignment accuracy by using the three methods, pure inertial navigation experiments were conducted after each aerial transfer alignment experiment. The results show that all the position errors by using the adaptive compensation H∞ filter were the smallest in the seven groups of aerial transfer alignment experiments, which verifies that the transfer alignment accuracy of the adaptive compensation H∞ filter was the highest among the three methods. It verifies the superiority of the proposed method. This research provides a new idea for SINS’ transfer alignment under a complex dynamic environment. Further research will focus on the implementation of the practical application in airborne distributed POS to validate the performance of this method.

## Figures and Tables

**Table 1:** The velocity errors (m/s) after 10 minutes’ pure inertial navigation.

Method	East Velocity Error	North Velocity Error	Velocity Error
Kalman Filter-CP	−2.17	2.58	3.38
H∞ Filter-CP	−1.11	1.93	2.26
A–C H∞ Filter-CP	−0.24	1.46	1.51

**Table 2:** The position errors (m) after 10 minutes’ pure inertial navigation.

Method	East Position Error	North Position Error	Position Error
Kalman Filter-CP	673.12	531.41	857.62
H∞ Filter-CP	433.85	362.93	565.38
A–C H∞ Filter-CP	226.07	231.56	323.69

**Table 3:** The position errors (m) in seven groups of aerial experiment.

Method	Group 1	Group 2	Group 3	Group 4	Group 5	Group 6	Group 7
Kalman Filter-CP	857.62	798.60	1084.35	509.24	1413.57	917.93	716.32
H∞ Filter-CP	565.38	419.71	792.53	311.57	1005.36	549.29	492.64
A–C H∞ Filter-CP	323.69	210.35	382.31	143.69	416.38	277.64	217.56
